# Adaptation of the Rey Auditory Verbal Learning Test and Logical Memory Subtest from the Wechsler Memory Scales – 3rd Edition to assess accelerated long‐term forgetting in adults with epilepsy

**DOI:** 10.1002/epd2.70084

**Published:** 2025-08-14

**Authors:** Amélie Landry, Isabelle Rouleau, Emma Colucci, Dang Khoa Nguyen, Olivier Boucher

**Affiliations:** ^1^ Département de Psychologie Université du Québec à Montréal (UQAM) Montréal Québec Canada; ^2^ Centre de Recherche du Centre Hospitalier de l'Université de Montréal (CRCHUM) Montréal Québec Canada; ^3^ Département de Neurosciences, Faculté de Médecine Université de Montréal Montréal Québec Canada; ^4^ Département de Psychologie Université de Montréal Montréal Québec Canada; ^5^ Service de Psychologie, Centre Hospitalier de L'université de Montréal (CHUM) Montréal Québec Canada

**Keywords:** accelerated long‐term forgetting, long‐term memory, neuropsychological tests, normative data, temporal lobe epilepsy

## Abstract

**Objective:**

The present study provides normative data for adapted versions of the Rey Auditory Verbal Learning Test (RAVLT) and the Logical Memory subtest from the Wechsler Memory Scales – 3rd edition (WMS‐III‐LM), involving both recall and recognition procedures after a 2‐week delay to assess accelerated long‐term forgetting (ALF). The study also aims to achieve a clinical validation of these tests in a group of people with epilepsy (PWE).

**Methods:**

A total of 124 (18–55 years old) healthy participants and 30 PWE undergoing presurgical monitoring for drug‐resistant seizures completed these tasks. Associations between memory performance and sociodemographic, neuropsychological function, and testing factors were examined among healthy participants. Memory performance was compared between healthy and PWE groups, with special attention to forgetting rates over 2 weeks as a measure of long‐term consolidation.

**Results:**

Contrarily to raw recall and recognition performance, forgetting rates over 2 weeks were not significantly modulated by age or sex. As expected, higher forgetting rates and a greater prevalence of ALF were found among PWE compared with healthy participants on both tests.

**Significance:**

This study offers useful normative data to assess ALF in PWE and provides clinical evidence that our adapted tests can identify long‐term consolidation impairments in this population.

**Key points**

- RAVLT and WMS‐III Logical Memory were adapted to assess ALF.
- Normative data were obtained from 124 adults.
- These adaptations are sensitive to ALF in PWE.

## 1 INTRODUCTION

Traditional memory theories suggest that long‐term memory consolidation occurs within the first 30 min after learning,^1^ though the entire process, including reconsolidation, can take years.^2^ Standard neuropsychological tests typically use a 20–30‐min delay to assess long‐term memory, effectively detecting impairments in various neurological conditions.^3^, ^4^, ^5^, ^6^ However, in some clinical conditions, such as temporal lobe epilepsy, the forgetting curve steepens beyond this timeframe, a phenomenon known as accelerated long‐term forgetting (ALF).^7^ ALF is marked by significant later forgetting of material initially learned and retained normally.^3^ While extensively studied in temporal lobe epilepsy,^8^ it has also been observed in extratemporal epilepsy,^9^ generalized epilepsy,^10^, ^11^ traumatic brain injury,^5^ Alzheimer's (both presymptomatic and symptomatic),^12^, ^13^ strokes,^14^ transient ischemic attacks,^14^ limbic encephalitis,^15^ and aging.^16^ However, ALF is not consistently present in all cases of epilepsy.^17^, ^18^

Improper assessment of ALF may explain the weak correlation between memory performance on standard tests and subjective complaints in conditions like focal epilepsy.^19^ A study of temporal lobe epilepsy patients with memory complaints found significant impairments when reassessed after a 3‐week delay, despite normal performance on standard tests.^20^ Other studies have shown stronger correlations between memory complaints and performance with longer recall delays,^8^ suggesting that extending the assessment delay improves sensitivity to memory deficits missed by traditional tests.^8^

There is no consensus on the best way to assess ALF, but some authors suggest adding a recall procedure after a longer delay to standard neuropsychological tests,^21^, ^22^ as it is practical in clinical settings. While many tests originally include a “yes/no” recognition trial after delayed recall, most ALF adaptations remove this trial to avoid re‐presenting the material.^23^ However, by being less affected by executive processes,^3^ recognition procedures may provide a better assessment of how information is encoded and consolidated.^24^

The optimal timeframe to assess ALF remains debated, but several studies suggest that a 1‐week delay is sufficient to detect ALF.^10^, ^23^, ^25^, ^26^, ^27^ Furthermore, a delay of at least 1 week improves the correspondence between subjective and objective memory impairments,^28^ highlighting the importance of extending the assessment beyond the typical 30‐min delay.

Although memory functions are thoroughly examined in presurgical evaluations for focal epilepsy, there are no solid normative data for measuring ALF, or those available lack recognition procedures, limiting the ability to distinguish between consolidation and retrieval deficits. This study adapted two existing neuropsychological tests to assess ALF by adding recall and recognition trials after a 2‐week delay. The first part provides normative data from healthy participants. The second part examines the tests' ability to detect ALF in people with epilepsy (PWE) with drug‐resistant seizures undergoing presurgical monitoring.

## 2 MATERIALS AND METHODS

### 2.1 Participants

For the first part of the study, a total of 126 healthy participants (63 women) aged from 18 to 55 years were recruited through ads in our medical center and its social media, ensuring equal distribution by sex, age, and education. This age range was chosen to reflect that of epilepsy patients referred for a presurgical assessment in the monitoring unit. Before assessment, potential participants completed a questionnaire to confirm they did not meet exclusion criteria, including neurological (including previous seizure) or psychiatric (e.g., chronic mental illness such as schizophrenia or bipolar disorder, intellectual disability (IQ < 70), or autism spectrum disorder, stroke, moderate or severe traumatic brain injury, or attention deficit disorder with or without hyperactivity or a similar disorder or doubts about the possibility of having such a disorder) history. Excluding those with suspected attention deficit disorder helped prevent participants from seeking a low‐cost neuropsychological assessment. Two men were excluded after testing was initiated—one due to a concussion after the first session and the other due to invalid performance, that is, very poor outcomes (<2.0 SD below the mean) in tasks assessing non‐verbal reasoning and language abilities, and questionable efforts to complete all tasks (i.e., responding too quickly for the answers to be genuine and behavior suggested a lack of engagement with the stimuli, such as not thoroughly observing all presented options before providing an answer). Thus, 124 healthy participants completed the study.

For the second part of the study, 31 PWE (18–55 years) referred for presurgical neuropsychological assessment due to suspected focal epilepsy with drug‐resistant seizures were recruited. PWE participants had to be fluent in French or English, have a confirmed epilepsy diagnosis, and have no other neurological (e.g., stroke, moderate or severe head trauma, neurodegenerative disorder) or psychiatric (e.g., chronic mental illness such as schizophrenia or bipolar disorder, intellectual disability (IQ < 70), or autism spectrum disorder) history. One participant did not complete the second assessment; hence, the total sample is composed of 30 individuals (18 women). Seventy percent of the PWE sample were recruited and completed their first assessment during their stay at the epilepsy monitoring unit; the others were tested in the outpatient clinic, and two were tested remotely. For the second assessment, 13% were tested again at the monitoring unit, four participants at the outpatient clinic, and the rest were assessed remotely. Data on demographics, neuropsychological function, and ALF assessment for study participants and epilepsy‐related data for PWE are shown in Table 1. No PWE experienced a seizure involving an altered state of consciousness shortly before or during the assessment, and the neuropsychological assessment was not conducted if the patient showed signs of post‐ictal impairments due to a recent seizure. Between the two assessments, 7 (23.3%) PWE changed antiseizure medication, while for 9 (30%) PWE, only the dosage was adjusted.

**TABLE 1:** Demographic, neuropsychological, and epilepsy‐related variables.

	HC (*n* = 124)			PWE (*n* = 30)			HC vs. PWE		
	Mean (SD)	Range	%	Mean (SD)	Range	%	*t*‐value /χ^2^	Effect size	*p‐*value
Demographics									
Age (years)	35.0 (11.2)	18–55		33.9 (9.6)	18–54		.21	−.09	.65
Education (years)	14.9 (2.1)	11–18		14.0 (2.5)	9–21		3.59	−.39	.06
Gender (% women)			50.8			60.0	.68	.07	.41
Ethnicity (% Caucasians)			75.8			76.7	.01	.01	.92
Neuropsychological assessment									
BNT total score (/30)	28.5 ± 2.2	17–30		27.5 (2.5)	20–30		4.27	−.42	.04*
WAIS‐IV‐MR scaled score (/19)	11.6 ± 2.8	4–18		10.3 (2.9)	6–19		5.67	−.48	.02*
Delay (days)	13.5 ± 2.9	7–21		13.9 (2.5)	7–19		.60	.16	.44
Location of epileptic focus									
Probable mesio‐temporal (%)			–			43.3	**–**		**–**
Other focus (%)			–			53.4	**–**		**–**
Generalized epilepsy (%)			–			3.3	**–**		**–**
Antiseizure medication									
No medication (%)			–			3.3	**–**		**–**
1 medication (%)			–			16.7	**–**		**–**
2 medications (%)			–			50.0	**–**		**–**
≥3 medications (%)			–			30.0	**–**		**–**

*Note*: *t*‐tests were done for continuous variables and chi‐squared for categorical variables. For the effect size, Φ was used for Chi‐squared and Cohen's *d* for the *t*‐tests. *p < .05.

### 2.2 Procedures

Participants completed two assessment sessions, either in‐person or remotely (via Microsoft Teams). Previous studies show that episodic memory performance is not significantly affected by assessment mode.^29^, ^30^, ^31^, ^32^ In the healthy control (HC) group, 26.6% had both sessions in‐person, 65.3% remotely, and 8.1% had the first in‐person and the second remotely. For this group, data collection took place from January 24, 2022, to September 8, 2023. In the PWE sample, 17% had both assessments in‐person, one participant had both assessments remotely, and the rest had the first in‐person and the second remotely. For this group, data collection occurred between June 14, 2022, and December 20, 2024. The first session (90–120 min) included tests of expressive language, logical reasoning, and memory. The second session (≈ 30 min) occurred about 14 days later (range: 7–14), depending on participants' availability, with long‐delay recall and recognition trials. Participants were instructed not to discuss or practice the learned material between sessions, without being told about the nature of the second session. All assessments were conducted in French. The time (i.e., morning vs. afternoon) of each assessment was not standardized and depended solely on the participant's availability. All participants (HC and PWE) provided their informed consent before the first assessment after confirming that they met the inclusion criteria and no exclusion criteria were present and received financial compensation of $50 for their participation. The project was approved by the CHUM Research Center Ethics Committee (approval number: 2019‐7770, 18.020).

### 2.3 Materials

#### 2.3.1 Memory tests

##### Rey auditory verbal learning test (RAVLT)^33^

The examiner reads a 15‐word list (List A), and the participant recalls as many words as possible over five learning trials. This is followed by a one‐trial recall of a 15‐word distractor list (List B). Immediate recall of List A is then performed, followed by delayed recall 30 min later. A yes/no recognition trial with 50 words (15 from List A, 15 from List B, and 20 new distractors) follows, where the participant indicates whether each word was in List A. A recognition accuracy score is calculated: “([correct hits – ([false alarms/35] × 15)]/15) × 100”. Recall and recognition procedures are repeated 2 weeks later with different distractors, except for those from List B (see Data S1).

##### Logical memory subtest – Wechsler memory scales – 3rd edition (WMS‐III‐LM)^34^

The examiner reads Story A for immediate recall, followed by Story B, read twice each time with immediate recall. After 30 min, delayed recall is completed. Delayed recall for both stories is followed by a cued recall task with specific questions (13 for Story A, 14 for Story B). If a participant cannot answer or gives a wrong answer, a three‐choice recognition trial is given. Incorrect responses involving a distractor option are replaced with a new option. No feedback is provided. One point is awarded for each correct answer in the cued recall questions (cued recall score), and another score is calculated by adding points for correct responses in the forced‐choice recognition task based on the cued recall (forced‐choice recognition score). Recall and recognition are repeated 2 weeks later, with new distractors introduced in the recognition trials (see Data S1 for details).

#### 2.3.2 Complementary neuropsychological testing

##### Boston naming test (BNT)^35^

An abbreviated version (30 items) of the BNT adapted for Franco‐Quebecers was completed by the participants^36^ to assess expressive language. Participants are asked to name an illustrated common object or an animal, with semantic and phonemic cues provided if needed.

##### Matrix reasoning subtest from the Wechsler adult intelligence scale 4th edition (WAIS‐IV‐MR)^37^

The subtest is a problem‐solving task involving abstract drawings. The participant must use logical deduction to identify which of the five choices completes the pattern formed by a group of stimuli. The latter subtest is used as it is strongly associated with fluid intelligence/reasoning.^38^ Scaled scores were obtained using the Canadian norms.^37^

### 2.4 Statistical analyses

Variable distribution was examined within the HC group. The influence of sociodemographic, neuropsychological function (BNT and WAIS‐IV‐MR), and assessment [testing mode (coded as 1: both assessments remotely, 2: both assessments in‐person, 3: one assessment in‐person and another remotely) and assessment delay] factors on memory performance was examined using Pearson correlation and ANOVAs. Mean scores (± SD) for memory tests were computed to establish normative data. The elbow method identified the optimal number of age categories, and the K‐means algorithm clustered individuals based on memory scores. Linear regressions were used to calculate *Z*‐scores for memory, adjusting for sociodemographic variables. Variables that were following a non‐linear relationship (i.e., quadratic polynomials) were adjusted in the equation. Percentiles (5th, 16th, and 25th, as suggested by Guilmette and colleagues^39^ to label the performance test scores) were computed for non‐normally distributed scores or where variance was too high (e.g., making it impossible to obtain a score ≥2 SD from the mean). Forgetting rates were calculated as:

$$\text{Forgetting rate}=\frac{\text{score}\;\mathrm{at}\;30\;\text{minutes}-\text{score}\;\mathrm{at}\;\mathrm{two}\;\text{weeks}}{\text{score}\;\mathrm{at}\;30\;\text{minutes}}\times 100.$$

For the PWE group, memory scores were converted into *Z*‐scores using normative data from the HC. Impaired performance was defined as a *Z*‐score ≤ −1.5 (≥1.5 for forgetting rate). ALF was defined by normal performance on the 30‐min delay (*Z* ≥ −1.5) and impaired forgetting rate at the 2‐week delay (*Z* ≥ 1.5), as similarly done by Miller and colleagues.^23^ Forgetting rates and the prevalence of ALF were compared between PWE and HC using ANOVA and chi‐square tests, respectively. Task conditions were compared, after all scores were brought back over 100, using mixed ANOVAs, with time as a within‐subject factor and group as a between‐subject factor, controlling for sex and age. When required, post hoc Tukey HSD tests were performed to examine differences between groups (ALF+, ALF−, HC). Exploratory t‐tests were conducted to examine differences in forgetting rates between patients with mesio‐temporal lobe epilepsy (mTLE) and those with non‐mTLE.

All analyses were performed using IBM SPSS (version 28) with a significance level of *p* < .05.

## 3 RESULTS

### 3.1 Association with sociodemographic variables

Memory scores that deviated from normal distribution include RAVLT 30‐min and 2‐week recognition hits, false recognitions, recognition score (i.e., % correct answers), and forgetting rate on recognition trials; WMS‐III‐LM 2‐week forced‐choice recognition for both stories, as well as forgetting rates on cued recall and forced‐choice recognition trials. Distributions for these variables at 2 weeks and forgetting rates are shown in Figures S1A (RAVLT) and S1B (WMS‐III‐LM) in the Data S1.

Age and sex were significantly associated with most memory scores, with younger participants and women performing better, though they were not associated with forgetting rates (see Table S1 for correlations between sociodemographic variables and memory performance). Higher education was associated with better WMS‐III‐LM scores, but not with RAVLT scores or forgetting rates. Ethnicity was not associated with memory scores for either test. No significant correlation was found between memory scores and the BNT. For the WAIS‐IV‐MR, no significant correlation was found (*p*s >.05) except for the RAVLT recognition forgetting rate (*ρ* = −.189, *p* = .035), WMS‐III‐LM immediate recall (*ρ* = .198, *p* = .028), and 30‐min delayed forced‐choice recognition (*ρ* = .183, *p* = .042). A longer delay between assessments was not linked to raw performance (*ps* >.05) but was associated with higher forgetting rates on the RAVLT (recall: *ρ* = .26, *p* = .004; recognition: *ρ* = .18, *p* = .04) and WMS‐III‐LM (recall: *ρ* = .35, *p* < .001; cued recall: *ρ* = .22, *p* = .01; forced‐choice recognition: *ρ* = .19, *p* = .04). Testing mode did not influence memory performance (*p*s >.05).

### 3.2 Normative data for the RAVLT and WMS‐III‐LM

Using the K‐means algorithm, the age variable was divided into three groups: 18–29, 30–41, and 42–55. Normative data for the RAVLT (Table 2) and WMS‐III‐LM (Table 3) were stratified by sex and age group. Additional WMS‐III‐LM normative data are reported in Table S2. Memory scores with non‐normal distribution or high variance (i.e., impossible to deviate by at least 2 SD below the calculated mean) are presented in Table 4. Scaled scores for WMS‐III‐LM immediate recall were: men 18–29 years = 10.6 ± 2.2, 30–41 years = 10.6 ± 3.5, 42–55 years = 9.9 ± 2.2; women: 18–29 years = 12.1 ± 2.9, 30–41 years = 11.6 ± 2.8, 42–55 years = 11.1 ± 3.2. Scaled scores for WMS‐III‐LM 30‐min delayed recall were: men 18–29 years = 10.7 ± 2.7, 30–41 years = 10.6 ± 3.6, 42–55 years = 9.3 ± 2.7; women 18–29 years = 12.1 ± 3.4, 30–41 years = 11.1 ± 2.7, 42–55 years = 11.7 ± 3.3.

**TABLE 2:** Mean (SD) RAVLT performance among the health control participants.

RAVLT score	Men (*n* = 61)			Women (*n* = 63)		
	18–29 (*n* = 21)	30–41 (*n* = 21)	42–55 (*n* = 19)	18–29 (*n* = 23)	30–41 (*n* = 18)	42–55 (*n* = 22)
Trial 1 (/15)	6.55 (1.73)	5.95 (1.96)	5.00 (1.73)	6.56 (1.81)	6.83 (1.98)	6.29 (1.98)
Trial 2 (/15)	10.15 (1.90)	8.26 (2.86)	8.71 (2.13)	10.04 (2.34)	9.94 (2.26)	9.24 (2.84)
Trial 3 (/15)	11.90 (1.94)	10.58 (2.65)	10.43 (1.89)	12.20 (2.18)	11.83 (1.65)	11.52 (1.99)
Trial 4 (/15)	13.00 (1.69)	11.37 (2.03)	10.90 (1.87)	13.04 (2.11)	12.83 (1.69)	12.90 (1.87)
Trial 5 (/15)	13.20 (1.58)	12.47 (2.17)	11.86 (1.77)	13.44 (1.42)	12.94 (1.59)	12.81 (1.89)
Ʃ Trials 1–5 (/75)	54.80 (6.53)	48.63 (10.40)	46.90 (7.66)	55.28 (8.19)	54.39 (8.19)	52.76 (8.20)
List B (/15)	6.90 (1.94)	4.63 (1.74)	4.24 (1.48)	7.00 (3.14)	6.00 (2.22)	5.38 (1.69)
Immediate recall (/15)	11.80 (2.29)	11.16 (2.36)	9.33 (2.97)	12.32 (2.34)	12.06 (2.36)	11.29 (2.60)
30‐min delayed recall (/15)	11.90 (1.86)	10.84 (2.67)	8.67 (3.24)	12.56 (2.08)	11.61 (2.81)	11.71 (2.26)
30‐min recognition						
Correct hits (/15)^b^	14.75 (.55)	14.53 (.77)	14.00 (1.10)	14.56 (.71)	14.61 (.70)	14.33 (1.02)
False positives (/35)^b^	.40 (.75)	3.21 (3.61)	2.95 (4.11)	.32 (1.07)	.72 (1.45)	2.00 (2.61)
Recognition score (/100)^a^, ^b^	97.19 (4.25)	87.66 (13.09)	84.90 (16.63)	96.15 (6.73)	95.34 (5.84)	89.84 (10.44)
2‐week delayed recall (/15)	6.15 (2.23)	5.11 (3.73)^c^	4.19 (2.50)^c^	6.56 (3.34)^c^	6.06 (3.37)^c^	5.29 (2.69)^c^
2‐week recognition						
Correct hits (/15)^b^	12.80 (1.47)	12.95 (2.88)	12.86 (1.68)	13.60 (1.00)	13.72 (1.53)	13.57 (1.21)
False positives (/35)^b^	2.35 (2.66)	6.00 (4.50)	6.38 (5.79)	2.12 (2.05)	4.83 (5.49)	5.24 (3.46)
Recognition score (/100)^a^, ^b^	78.62 (13.09)	69.17 (25.40)	67.48 (18.19)	84.61 (9.79)	77.67 (18.24)	75.51 (13.04)

Forgetting rate	Delay (days)		
	7–13 (*n* = 47)	14 (*n* = 49)	15–21 (*n* = 28)
Recall (/100)	43.77 (19.53)	56.12 (23.04)	54.82 (23.86)
Recognition hits (/100)^b^	11.44 (11.00)	14.48 (12.15)	20.85 (17.55)
Recognition score (/100)^b^	13.67 (12.75)	17.70 (14.25)	24.48 (21.12)

^a^ Recognition scores were computed using the following formula: ([correct hits – ([false alarms/35]× 15)] / 15) × 100.

^b^ Variables non‐normally distributed.

^c^ Variables for which scores cannot get at least 2 SD below the average.

**TABLE 3:** Mean (SD) WMS‐III‐LM performance among the healthy control participants.

WMS‐III‐LM score	Men (*n* = 61)			Women (*n* = 63)		
	18–29 (*n* = 21)	30–41 (*n* = 21)	42–55 (*n* = 19)	18–29 (*n* = 23)	30–41 (*n* = 18)	42–55 (*n* = 22)
Total recall (A + B1 + B2) (/75)	42.55 (7.82)	44.05 (11.59)	38.29 (8.00)	48.12 (8.86)	47.56 (8.73)	42.24 (10.93)
30‐min delayed recall (A + B) (/50)	27.20 (6.63)	26.84 (8.47)	22.19 (6.41)	30.04 (7.29)	30.22 (6.22)	27.33 (7.83)
30‐min recognition						
Cued recall A + B (/27)	17.65 (4.33)	18.68 (4.85)	16.48 (3.87)	20.20 (3.71)	20.44 (2.71)	18.86 (4.61)
Forced‐choice story A + B (/27)	24.20 (2.51)	24.37 (2.22)	23.86 (2.03)	24.76 (2.17)	25.22 (1.80)	24.81 (1.75)
2‐week delayed recall (A + B) (/50)	19.50 (6.15)	19.05 (9.28)	17.14 (6.58)	22.64 (7.11)	20.67 (6.61)	20.95 (7.30)
2‐week recognition						
Cued recall A + B (/27)	15.10 (4.46)	15.42 (5.48)	13.81 (3.68)	17.32 (3.87)	16.89 (3.48)	17.43 (5.94)
Forced‐choice story A + B (/27)^a^	23.15 (2.25)	23.21 (3.46)	22.81 (2.29)	24.72 (1.75)	23.89 (2.11)	24.48 (3.11)

Forgetting rate	Delay (days)		
	7–13 (*n* = 47)	14 (*n* = 49)	15–21 (*n* = 28)
Recall (/100)	17.83 (20.91)	28.45 (17.39)	36.12 (19.31)
Cued recall (/100)^a^	9.44 (19.19)	14.40 (17.09)	20.01 (24.38)
Forced‐choice score (/100)^a^	1.16 (9.89)	3.32 (10.86)	6.26 (7.95)

^a^ Variables non‐normally distributed.

**TABLE 4:** Percentiles (5, 16, 25) for the RAVLT and WMS‐III‐LM's scores that are not normally distributed among healthy control participants.

RAVLT score	Men (*n* = 61)			Women (*n* = 63)		
	5	16	25	5	16	25
30‐minutes recognition						
Correct hits (/15)	12.05	14.00	14.00	13.00	14.00	14.00
False positives (/35)	9.95	6.00	3.00	4.75	3.00	1.00
Recognition score (/100)	60.05	76.19	86.90	73.10	85.14	89.05
2‐week delayed recall (/15)	1.00	2.00	3.00	1.00	3.00	4.00
2‐week recognition						
Correct hits (/15)	9.10	11.00	12.00	11.25	12.00	13.00
False positives (/35)	15.00	10.00	8.00	10.00	7.00	6.00
Recognition score (/100)	30.48	50.48	64.76	54.76	66.10	69.52

	Total sample (*N* = 124)		
	5	16	25
Forgetting rate			
Recognition hits (/100)	26.67	20.00	13.33
Recognition score (/100)	48.54	32.65	25.99

WMS‐III‐LM scores	Men (*n* = 61)			Women (*n* = 63)		
	5	16	25	5	16	25
2‐week recognition						
Forced‐choice (A + B) (/27)	18.05	20.00	21.25	19.50	22.40	23.25

	Total sample (*N* = 124)		
	5	16	25
Forgetting rate			
Cued recall score (/100)	25.00	33.33	49.26
Forced‐choice recognition score (/100)	8.00	11.54	20.83

Linear regression results and equations to calculate adjusted *Z*‐scores [calculated as: (Actual score – Expected score)/$\sqrt{\left(\text{mean square residual}\right)}$] for both memory tasks, based on regression models for normally distributed memory variables are in Table S3. For all RAVLT variables (except forgetting rates) and immediate/30‐min recalls of the WMS‐III‐LM, the best predictive model included sex and age. No significant interactions were found among the variables of interest. For other WMS‐III‐LM variables, sex was the sole predictor. Forgetting rates for both tests were best predicted by the delay between assessment sessions.

### 3.3 Prevalence of ALF in PWE

Table 5 presents mean forgetting rates between 30 min and 2 weeks across memory tests for PWE and HC groups, along with ALF prevalence. PWE showed significantly higher forgetting rates than HC on all measures, except for WMS‐III‐LM forced‐choice recognition. ALF was also more common in PWE on RAVLT recognition hits, WMS‐III‐LM recall, and WMS‐III‐LM cued recall.

**TABLE 5:** Forgetting rates and frequency of ALF in both groups for both tasks.

	Mean (SD) forgetting rates		Comparisons with HC for forgetting rates		% impairment in forgetting rate		% ALF		Comparisons with HC for ALF	
	PWE	HC	*F*‐value	*p*‐value	PWE	HC	PWE	HC	χ^2^ value	*p*
RAVLT										
Recall	69.49 (20.69)	51.14 (22.55)	16.48	<.001**	23.3	7.3	13.3	5.7	2.15	.14
Recognition (hits)	18.26 (16.09)	8.21 (11.55)	15.48	<.001**	20.0	1.6	16.7	1.6	12.62	<.001**
Recognition (% correct)	30.13 (18.08)	17.71 (15.96)	13.88	<.001**	23.3	5.7	13.3	5.7	2.15	.14
WMS‐III‐LM										
Recall	40.23 (27.59)	26.16 (20.36)	9.94	.02**	30.0	7.3	23.3	6.5	7.83	.005**
Cued recall	31.32 (20.46)	13.78 (19.96)	18.47	<.001**	23.3	6.5	23.3	6.5	7.83	.005**
Forced‐choice	4.87 (12.66)	2.96 (9.70)	.82	.822	10.0	8.9	10.0	7.3	.25	.62

*Note*: The forgetting rate represents the percentage of total information forgotten between the 30‐min and 2‐week delays. % Impairment in forgetting rate: % of individuals in the sample with a *Z* ≥ 1.5 for their forgetting rate. % ALF: % of individuals in the sample with ALF, defined as having a “normal” performance (*Z* ≥ −1.5) at the 30‐minute delay and an impaired forgetting rate (*Z* ≥ 1.5) between the 30‐minute and the 2‐week delay. *p < .05; **p < .01.

Abbreviations: HC, healthy controls; PWE, people with epilepsy.

The mixed ANOVA revealed a significant group effect, with PWE scoring lower than HC on all tests and procedures (RAVLT: recall *F*_1, 150_ = 13.91, *p* = <.001, *η* ^2^ = .084; recognition *F*_1, 150_ = 5.58, *p* = .019, *η* ^2^ = .036; WMS‐III‐LM: recall *F*_1,150_ = 4.26, *p* = .04, *η* ^2^ = .028; cued recall *F*_1, 150_ = 57.62, *p* = <.001, *η* ^2^ = .278; forced‐choice recognition *F*_1, 150_ = 325.06, *p* = <.001, *η* ^2^ = .684). A significant time effect indicated decreased memory performance over time for both tests (RAVLT: recall *F*_1.47, 221.18_ = 23.52, *p* = <.001, *η* ^2^ = .136; recognition *F*_1, 150_ = 26.08, *p* = <.001, *η* ^2^ = .148; WMS‐III‐LM: recall *F*_1.55, 232.09_ = 67.66, *p* = <.001, *η* ^2^ = .311; cued recall *F*_1, 150_ = 42.99, *p* = <.001, *η* ^2^ = .223; forced‐choice recognition *F*_1, 150_ = 76.17, *p* = <.001, *η* ^2^ = .337). A significant interaction between group and time was found for all recall and recognition trials [RAVLT recall (*F*_1.47, 221.18_ = 4.18, *p* = .03, *η* ^2^ = .027) and recognition (*F*_1, 150_ = 9.35, *p* = .003, *η* ^2^ = .059) and the WMS‐III‐LM cued recall (*F*_1, 150_ = 197.29, *p* = <.001, *η* ^2^ = .568), and forced‐choice recognition (*F*_1, 150_ = 777.85, *p* = <.001, *η* ^2^ = .838)] except WMS‐III‐LM recall (*F*_1.55, 232.09_ = 2.91, *p* = .07, *η* ^2^ = .019), for which the interaction fell short of statistical significance. All interactions indicated steeper forgetting slopes for PWE across all recall and recognition trials.

**FIGURE 1:**
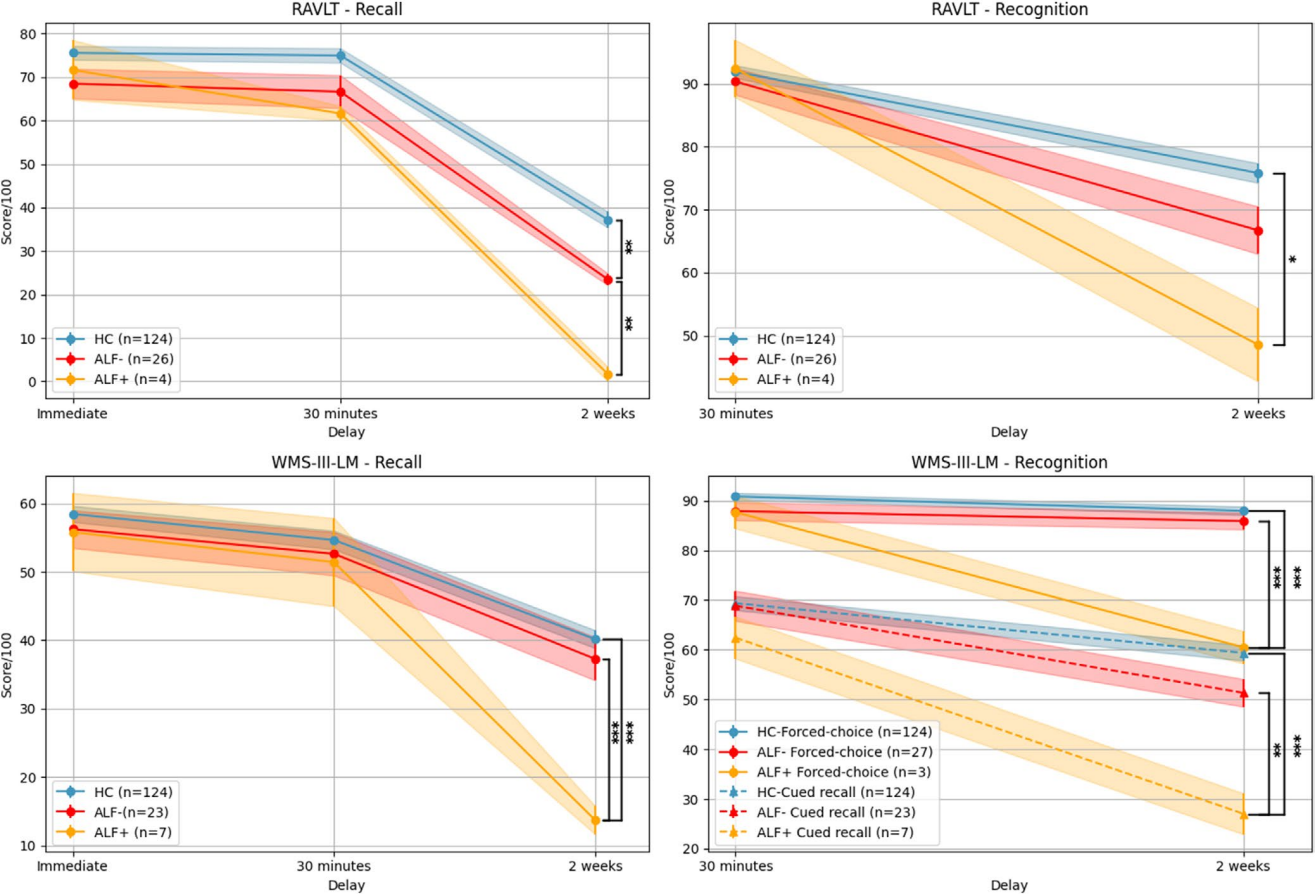
Scores out of 100 over time with standard error band for the RAVLT and the WMS‐III‐LM for people with epilepsy (PWE; *n* = 30) with ALF (ALF+) or without ALF (ALF−) and the healthy controls (HC; *n* = 124). The ‘*’ symbol indicates a statistically significant difference between groups at each time point, as determined by the mixed ANOVA; *p < .05, **p < .01, and ***p < .001.

The PWE group was further divided into those with (ALF+) and without ALF (ALF−). Post hoc analyses revealed that both ALF+ and ALF− groups exhibited greater forgetting over 2 weeks than HC on RAVLT recall. On RAVLT recognition, ALF+ forgot faster than HC, while ALF‐ did not differ from the other groups. In the WMS‐III‐LM, ALF+ showed greater forgetting compared with both HC and ALF‐ on recall, cued recall, and forced‐choice recognition. Figure 1 displays scores (brought back over 100) at each time point, highlighting statistical differences between groups. On the RAVLT measures, 15% of patients with mTLE showed ALF for recall, 23% for recognition, and 15% for recognition hits. For the non‐mTLE patients, 12% showed ALF on all different RAVLT variables. On the WMS‐III‐LM variables, 31% of the mTLE group had ALF for recall, 31% for cued recall, and 15% for forced‐choice recognition. For the non‐mTLE patients, 18% showed ALF for recall and cued recall, and 5% for forced‐choice recognition. Following exploratory analysis, statistical comparisons between patients with mTLE and those with non‐mTLE regarding forgetting rates did not reach statistical significance on any measure (all *ps* >.05).

## 4 DISCUSSION

In this study, the RAVLT and the WMS‐III‐LM were adapted to evaluate ALF in PWE. In the first part of the study, normative data were collected from individuals aged 18–55, providing recall and recognition scores after a 2‐week delay, stratified by demographic and assessment variables. Raw scores varied by age and sex, but forgetting rates were unrelated to sociodemographics and correlated only with the delay before the second assessment. Linear regression models generated Z‐score equations correcting for relevant variables, creating a robust reference for ALF assessment. In the second part of the study, the adapted tasks were administered to 30 PWE undergoing presurgical evaluations for drug‐resistant seizures. ALF was significantly more prevalent in PWE based on the normative data from HC.

This study stands out from prior research on ALF normative data due to its larger sample size (e.g., *n* = 60 in prior study) and sociodemographically corrected norms.^23^ The superior verbal memory performance in women in comparison to men is consistent with the existing literature, emphasizing the need to consider sex or gender in test interpretation, for example,^40^, ^41^ Age‐related performance trends in our dataset are consistent with memory theories, showing recall performance declines with age, while recognition is less affected, as the different episodic memory processes age differently.^21^, ^42^ Free recall, reliant on frontal lobe functions like attention and executive processes, declines more rapidly, whereas recognition, linked to medial temporal lobe consolidation, remains more stable.^24^, ^42^ Education was not significantly associated with RAVLT performance, which is consistent with findings by Mitrushina and colleagues.^43^ However, it correlated with raw WMS‐III‐LM scores, but not forgetting rates. The sample's higher education level (68% with a degree vs. 42.5% in Quebec's population^44^) may explain why WMS‐III‐LM immediate and 30‐min recall scores are slightly higher than the original norms. However, the lack of association between education and key performance metrics supports the robustness of these normative data for ALF assessment.

We found that longer delays between assessment sessions (7–21 days) were linked to higher forgetting rates, emphasizing the importance of adhering to the 2‐week timeframe for optimal use of normative data; though some flexibility is acceptable. In contrast, the assessment modality (in‐person vs. remote; consistent vs. varied) did not significantly affect performance, consistent with prior studies.^45^, ^46^ Indeed, in a previous study, no differences were found between 69 face‐to‐face and 71 remote assessments, assessing memory, praxis, executive functions, language, and working memory,^44^ and another study showed similar remote and normative scores in 240 participants across verbal memory tests.^45^ These findings streamline clinical assessments and support tele‐neuropsychological practices.

Mean RAVLT scores for immediate and 30‐min delayed recall in our participants closely match those reported for similar age groups in prior studies,^41^ supporting the reliability of our normative data. Recall performance after a 2‐week delay aligns with studies using delays of 1 week (e.g., Hoefeijzers et al., *n* = 18, mean age = 68.3 years^25^; Visser et al., *n* = 18, mean age ≈ 41 years^27^) and longer intervals like three^25^ and 4 weeks (*n* = 59, mean age = 31.0^47^), with recognition trial performance also comparable across studies after delays of one^27^ and 3 weeks.^25^ Notably, some studies omitted recognition procedures after earlier assessments^47^ but retained them for longer delays^25^ or required additional learning trials for participants to reach a performance threshold.^25^ In our study, the 2‐week forgetting rate for recall trials (for 7–13 days: 43.8 ± 19.5) is higher than Miller and colleagues^23^ findings for a 7‐day delay (36.3 ± 21.8), likely due to the longer delay.

With regards to WMS‐III‐LM performance, the immediate and 30‐min delayed recall scores align with normative data for French and American populations,^34^, ^48^ and 2‐week recall scores match Bell^49^ findings for normal controls (*n* = 25; mean age = 35.0). Forgetting rates for recall trials (7–13 days: 17.8 ± 20.9) are lower than the results of Miller and colleagues^23^ after 1‐week delay (29.2 ± 20.6), possibly due to differences in Logical Memory subtest versions used (i.e., WMS‐III wherein story B is read twice in our study, and WMS‐IV in which stories A and B are both only read once in Miller and colleagues' study). Our study uniquely provides normative data for recognition trials of the WMS‐III‐LM after a long delay. The inclusion of cued recall offers a less variable alternative to recall trials, facilitating differentiation between normal and abnormal performance.

Unsurprisingly, forgetting rates were higher for recall than recognition trials on both the RAVLT and WMS‐III‐LM, suggesting that information may remain consolidated but be harder to recall spontaneously over time. As ALF is thought to involve consolidation deficits, including recognition procedures is essential,^25^ as recommended by Elliot and colleagues,^3^ to avoid overestimating ALF. Keeping recognition procedures helps to delve deeper into consolidation deficits associated with ALF,^25^, ^50^ rather than being altered by retrieval anomalies and acting as a reminder,^23^ which could compromise free recall.^26^ Also, assessing episodic memory after delays longer than the standard 30 min increases sensitivity to consolidation impairments.^22^, ^25^

While forgetting rates significantly distinguished the groups on all test variables except for WMS‐III‐LM forced‐choice recognition, ALF was significantly more prevalent in PWE compared with HC on RAVLT recognition (hits), WMS‐III‐LM recall, and WMS‐III cued recall. Excluding participants with impaired performance on the 30‐min delayed trial from those categorized as “ALF” may have contributed to underestimating ALF among PWE. Among RAVLT scores, recognition hits appear to be the most effective in distinguishing between PWE and HC for ALF. The prevalence of PWE and HC displaying ALF for the RAVLT recall is comparable to those reported by Miller and colleagues for a 1‐week delay (18% in PWE, 5% in HC).^51^ For the WMS‐III‐LM, recall and cued recall both distinguished the PWE and HC for ALF, whereas the forced‐choice recognition trial lacked sensitivity to detect ALF. Interestingly, ALF was found among PWE with mTLE, but also in those with non‐mTLE, which is congruent with previous observations.^8^, ^9^, ^23^ A larger sample is needed to examine the contribution of the epileptogenic zone location to ALF. Additionally, based on recent findings, more clinical variables should be considered, as slow‐wave sleep and interictal epileptiform activity may also contribute to ALF.^52^

Limitations to our study include the voluntary nature of participants, which potentially biases the sample toward individuals interested in or confident about their memory abilities. The HC sample, comprising highly educated urban participants aged 18–55, may limit generalizability to older, rural, or less‐educated populations. Additionally, participants were aware that the study involved two sessions, which might have led some to anticipate recall tasks in the second session. While Elliott and colleagues^3^ raised concerns about minimizing rehearsal during delays, this remains challenging to control in real‐world contexts. Also, the time of day when testing occurred (morning vs. afternoon) was not controlled in our study. This may have influenced the results, as cognitive performance shows slight changes throughout the day^53^; however, it reflects the practical constraints of neuropsychological assessments, which cannot always be scheduled at fixed times. Similarly, medication changes may have affected performance, but they are inherent to evaluating PWE in an epilepsy monitoring unit. While dosage adjustments are generally considered to have a limited impact compared with polytherapy,^54^ current evidence suggests that polytherapy itself is unlikely to be directly associated with ALF.^55^, ^56^, ^57^ Although some antiseizure medications can impair short‐term memory, there is no compelling evidence or clear mechanism to explain a specific effect on long‐term memory consolidation.^58^

## 5 CONCLUSION

This study provides valuable tools for assessing ALF in PWE, with test variables (e.g., false recognitions) that support clinicians' qualitative observations with quantitative data. The equations for calculating Z‐scores, corrected for sociodemographic variables through linear regression, help avoid “gap” effects when transitioning between age groups. The adapted RAVLT and WMS‐III‐LM tasks were shown to effectively detect ALF in PWE undergoing presurgical evaluation for drug‐resistant seizures. This study aims to encourage a more systematic assessment of long‐term consolidation processes in standard neuropsychological evaluations for PWE and potentially other clinical populations.

## AUTHOR CONTRIBUTIONS

**Amélie Landry**: Conceptualization, Formal analysis, Investigation, Visualization, Writing – Original Draft. **Isabelle Rouleau**: Conceptualization, Supervision, Writing – Review and Editing. **Emma Colucci:** Investigation, Writing – Review and Editing. **Dang Khoa Nguyen:** Funding acquisition, Resources, Writing – Review and Editing. **Olivier Boucher:** Conceptualization, Investigation, Supervision, Writing – Review and Editing.

## FUNDING INFORMATION

This research was funded by grants from the Natural Sciences and Engineering Research Council (NSERC) grant number: RGPIN‐2022‐04255 (DKN), by a master grant from the Savoy Foundation for Epilepsy, master and doctoral grants from the Canadian Institutes of Health Research (CIHR), and the Fonds de Recherche en Santé du Québec (FRQS) grant number: 328183 (AL), and by a salary support for health professionals grant from the FRQS (OB). DKN holds the Canada Research Chair in Epilepsy and Functional Anatomy of the Human Brain.

## CONFLICT OF INTEREST STATEMENT

There is no conflict of interest to disclose.

**Test yourself**

1. What is a potential drawback of only using traditional 20–30‐minute delays on episodic memory tests when assessing memory in people with epilepsy (PWE)?
  - These tests primarily assess working memory rather than long‐term memory
  - They fail to detect accelerated long‐term forgetting (ALF), which occurs over days rather than minutes
  - They are not sensitive to memory impairments in epilepsy and tend to produce false‐negatives
  - They confound memory deficits with executive dysfunction due to their high cognitive load
2. What methodological limitation of previous ALF assessments does this study aim to address?
  - The reliance on small normative sample without sociodemographic considerations
  - The failure to distinguish between recall and recognition performance
  - The overrepresentation of individuals with drug‐resistant epilepsy
  - A and B
  - All of the above
3. Which of the following best explains the rationale for including both recall and recognition trials in the adapted tasks?
  - To isolate the effects of consolidation deficits from retrieval impairments in ALF
  - To determine whether forgetting is due to proactive interference
  - To enhance the ecological validity of episodic memory assessments
  - To control for potential differences in attentional load between groups

*Answers may be found in the* *supporting information*

## Supporting information


Data S1.

Data S2.

## Data Availability

The data that support the findings of this study are available on request from the corresponding author. The data are not publicly available due to privacy or ethical restrictions.
