# Supplementary material for: Adaptation of the Rey Auditory Verbal Learning Test and Logical Memory Subtest from the Wechsler Memory Scales – 3rd Edition to assess accelerated long‐term forgetting in adults with epilepsy
Source: Epileptic Disord. 2025 Aug 14;27(6):1187–200. doi: 10.1002/epd2.70084 (PMC12747703; doi:10.1002/epd2.70084)
Supplement: Supplementary file 1 — Data S1. [file EPD2-27-1187-s002.docx]

**Supplementary Material**

**WMS-III-LM Recognition Procedure at 30 minutes – English Version**

| # Question | Spont. | Choice | # Question | | Spont. | Choice | |
| --- | --- | --- | --- | --- | --- | --- | --- |
| 1. What was the woman’s first name? |  |  | 8. How much money did they steal from her? | |  |  | |
| - Anna |  | 1 | - 56 $ | |  | 1 | |
| - Ashley |  | 0 | - 65 $ | |  | 0 | |
| - Angela |  | 0 | - 84 $ | |  | 0 | |
| 2. What was the woman’s last name? |  |  | 9. On which street did the robbery take place? | |  |  | |
| - Thompson |  | 1 | - St-Laurent | |  | 0 | |
| - Johnson |  | 0 | - Ste-Catherine | |  | 1 | |
| - Taylor |  | 0 | - St-Denis | |  | 0 | |
| 3. Where was she from? |  |  | 10. How many children did she have? | |  |  | |
| - Eastern Townships |  | 0 | - 0 child | |  | 0 | |
| - West Montreal |  | 1 | - 2 children | |  | 0 | |
| - Québec |  | 0 | - 4 children | |  | 1 | |
| 4. What kind of job did she have? |  |  | 11. What wasn’t paid? | |  |  | |
| - Waitress |  | 0 | - Her electricity bill | |  | 0 | |
| - Cleaning lady |  | 0 | - Her rent | |  | 1 | |
| - Cook |  | 1 | - Her cell phone | |  | 0 | |
| 5. Where did she work? |  |  | 12. How many days had it been since they had last eaten? | |  |  | |
| - In a hotel |  | 0 | - 1 day | |  | 0 | |
| - In a nursing home |  | 0 | - 2 days | |  | 1 | |
| - In a school |  | 1 | - 3 days | |  | 0 | |
| 6. What happened to her? |  |  | 13. What did the police do? | |  |  | |
| - She was robbed |  | 1 | - Drove her home | |  | 0 | |
| - She had an accident |  | 0 | - Arrested the thief | |  | 0 | |
| - She won a prize |  | 0 | - Took up a collection for her | |  | 1 | |
| 7. When was she robbed? |  |  |  | |  |  | |
| - The night before |  | 1 |  |  |  |  | |
| - Two days ago |  | 0 |  |  |  |  | |
| - Monday |  | 0 |  |  |  |  | |
|  |  |  | Total cued recall  = /13 | Total multiple choices = | | | /13 |

***Story A and B^1^***

| # Question | Spont. | Choice | # Question | | Spont. | Choice | |
| --- | --- | --- | --- | --- | --- | --- | --- |
| 1. What was the man’s first name? |  |  | 8. When was it supposed to start? | |  |  | |
| - Joe |  | 1 | - Around midnight | |  | 0 | |
| - John |  | 0 | - In 2 to 3 hours | |  | 1 | |
| - James |  | 0 | - Early in the morning | |  | 0 | |
| 2. What was the man’s last name? |  |  | 9. Until when was this special weather forecast? | |  |  | |
| - Smith |  | 1 | - Until the morning | |  | 1 | |
| - Simon |  | 0 | - Until Tuesday evening | |  | 0 | |
| - Stewart |  | 0 | - Until Wednesday | |  | 0 | |
| 3. Where did he live? |  |  | 10. How many inches of rain were expected? | |  |  | |
| - Boucherville |  | 0 | - 2 inches | |  | 0 | |
| - Ste-Julie |  | 0 | - 5 inches | |  | 1 | |
| - Sherbrooke |  | 1 | - 7 inches | |  | 0 | |
| 4. What day of the week was it? |  |  | 11. By how many degrees was the temperature supposed to drop? | |  |  | |
| - Friday |  | 0 | - 5 degrees | |  | 0 | |
| - Sunday |  | 0 | - 10 degrees | |  | 0 | |
| - Monday |  | 1 | - 15 degrees | |  | 1 | |
| 5. What time was it? |  |  | 12. Other than rain and a drop in temperature, what is forecasted? | |  |  | |
| - 5 pm |  | 0 | - Hail | |  | 1 | |
| - 6 pm |  | 1 | - Snow | |  | 0 | |
| - 8 pm |  | 0 | - Strong winds | |  | 0 | |
| 6. What did he do while watching television before the weather report? |  |  | 13. What did the man decide to do? | |  |  | |
| - Getting dressed |  | 1 | - Stay at home | |  | 1 | |
| - Washing the dishes |  | 0 | - Put his car in the garage | |  | 0 | |
| - Sitting in his chair |  | 0 | - Warn the neighbors | |  | 0 | |
| 7. What did the weather bulletin say? |  |  | 14. What did he watch on television? | |  |  | |
| - A thunderstorm |  | 1 | - A sports program | |  | 0 | |
| - A snowstorm |  | 0 | - The news | |  | 0 | |
| - High winds |  | 0 | - Old movies | |  | 1 | |
|  |  |  | Total cued recall  = /14 | Total multiple choices = | | | /14 |

*Note.* 1. The examiner must change an answer choice if the participant gives an incorrect response that is part of the answer choices.

| # Question | Spont. | Choix | # Question | | Spont. | Choix |
| --- | --- | --- | --- | --- | --- | --- |
| 1. Quel était le prénom de la femme ? |  |  | 8. Combien d’argent lui a-t-on volé ? | |  |  |
| - Jeanne |  | 1 | - 56 $ |  |  | 1 |
| - Jeannette |  | 0 | - 65 $ |  |  | 0 |
| - Jacynthe |  | 0 | - 84 $ |  |  | 0 |
| 2. Quel était le nom de famille de la femme ? |  |  | 9. Sur quelle rue a eu lieu le vol ? | |  |  |
| - Duval |  | 1 | - St-Laurent |  |  | 0 |
| - Laval |  | 0 | - Ste-Catherine |  |  | 1 |
| - Proulx |  | 0 | - St-Denis |  |  | 0 |
| 3. D’où venait-elle ? |  |  | 10. Combien avait-elle d’enfants ? | |  |  |
| - Canton de l’Est |  | 0 | - 0 enfant |  |  | 0 |
| - Montréal-Est |  | 1 | - 2 enfants |  |  | 0 |
| - Québec |  | 0 | - 4 enfants |  |  | 1 |
| 4. Que faisait-elle comme travail ? |  |  | 11. Qu’est-ce qui n’était pas payé ? | |  |  |
| - Serveuse |  | 0 | - Sa facture d’électricité |  |  | 0 |
| - Femme de ménage |  | 0 | - Son loyer |  |  | 1 |
| - Cuisinière |  | 1 | - Son cellulaire |  |  | 0 |
| 5. Où travaillait-elle ? |  |  | 12. Depuis combien de temps n’avaient-ils pas mangé ? |  |  |  |
| - Dans un hôtel |  | 0 | - 1 jour |  |  | 0 |
| - Dans un centre d’accueil |  | 0 | - 2 jours |  |  | 1 |
| - Dans une école |  | 1 | - 3 jours |  |  | 0 |
| 6. Que lui est-il arrivé ? |  |  | 13. Qu’on fait les policiers ? |  |  |  |
| - Elle a été volée |  | 1 | - Reconduit la femme chez elle | |  | 0 |
| - Elle a eu un accident |  | 0 | - Arrêté le voleur |  |  | 0 |
| - Elle a gagné un prix |  | 0 | - Donné de l’argent à la femme | |  | 1 |
| 7. Quand a-t-elle été volée ? |  |  |  | |  |  |
| - La veille |  | 1 |  |  |  |  |
| - Il y a 2 jours |  | 0 |  |  |  |  |
| - Lundi |  | 0 |  |  |  |  |
|  | | | Total rappel indicé  = /13 | Total choix  multiples = | | /13 |

**WMS-III-LM Recognition Procedure at 30 minutes – French Version**

***Story A and B^1^***

| # Question | Spont. | Choix | # Question | | Spont. | Choix |
| --- | --- | --- | --- | --- | --- | --- |
| 1. Quel était le prénom de l'homme ? |  |  | 8. Quand cela devait-il commencer ? | |  |  |
| - Pierre |  | 1 | - Vers minuit |  |  | 0 |
| - Patrick |  | 0 | - Dans 2 à 3 heures |  |  | 1 |
| - Paul |  | 0 | - Tôt le matin |  |  | 0 |
| 2. Quel était le nom de famille de l'homme? |  |  | 9. Jusqu'à quand cette météo spéciale était-elle prévue ? | |  |  |
| - Gagnon |  | 1 | - Jusqu'au matin |  |  | 1 |
| - Gagné |  | 0 | - Jusqu'à mardi soir |  |  | 0 |
| - Duguay |  | 0 | - Jusqu'à mercredi |  |  | 0 |
| 3. Où habitait-il ? |  |  | 10. Combien de cm de pluie étaient prévus ? | |  |  |
| - Boucherville |  | 0 | - 5 cm |  |  | 0 |
| - Ste-Julie |  | 0 | - 10 cm |  |  | 1 |
| - St-Hyacinthe |  | 1 | - 15 cm |  |  | 0 |
| 4. Quel jour de la semaine se passe l'histoire ? |  |  | 11. De combien de degrés chuterait la température ? | |  |  |
| - Vendredi |  | 0 | - 5 degrés |  |  | 0 |
| - Dimanche |  | 0 | - 10 degrés |  |  | 0 |
| - Lundi |  | 1 | - 15 degrés |  |  | 1 |
| 5. Quelle heure était-il ? |  |  | 12. Autre que la pluie et la baisse de température, qu'est-il annoncé ? | |  |  |
| - 5h00 |  | 0 | - De la grêle |  |  | 1 |
| - 6h00 |  | 1 | - De la neige |  |  | 0 |
| - 8h00 |  | 0 | - De forts vents |  |  | 0 |
| 6. Que faisait-il en regardant la télévision avant le bulletin météo ? |  |  | 13. Que décida de faire l’homme ? | |  |  |
| - Il s'habillait |  | 1 | - Rester à la maison |  |  | 1 |
| - Il faisait la vaisselle |  | 0 | - Rentrer sa voiture dans le garage | |  | 0 |
| - Il était assis dans son fauteuil |  | 0 | - Avertir ses voisins |  |  | 0 |
| 7. Qu'annonçait le bulletin météo ? |  |  | 14. Qu'a-t-il regardé à la télévision ? | |  |  |
| - Un orage |  | 1 | - Une émission de sport |  |  | 0 |
| - Une tempête de neige |  | 0 | - Les nouvelles |  |  | 0 |
| - Des vents violents |  | 0 | - Un film |  |  | 1 |
|  | | | Total rappel indicé  = /14 | Total choix  multiples = | | /14 |

*Note.* 1. The examiner must change an answer choice if the participant gives an incorrect response that is part of the answer choices.

**WMS-III-LM Recognition Procedure at Two Weeks – English Version**

***Story A and B^1^***

| # Question | Spont. | Choice | # Question | | Spont. | Choice | |
| --- | --- | --- | --- | --- | --- | --- | --- |
| 1. What was the woman’s first name? |  |  | 8. How much money did they steal from her? | |  |  | |
| - Amy |  | 0 | - 42 $ | |  | 0 | |
| - Anna |  | 1 | - 56 $ | |  | 1 | |
| - April |  | 0 | - 75 $ | |  | 0 | |
| 2. What was the woman’s last name? |  |  | 9. On which street did the robbery take place? | |  |  | |
| - Anderson |  | 0 | - St-Joseph | |  | 0 | |
| - Tucker |  | 0 | - Ste-Catherine | |  | 1 | |
| - Thompson |  | 1 | - Sherbrooke | |  | 0 | |
| 3. Where was she from? |  |  | 10. How many children did she have? | |  |  | |
| - West Montreal |  | 1 | - 1 child | |  | 0 | |
| - North Montreal |  | 0 | - 3 children | |  | 0 | |
| - Longueuil |  | 0 | - 4 children | |  | 1 | |
| 4. What kind of job did she have? |  |  | 11. What wasn’t paid? | |  |  | |
| - Teacher |  | 0 | - Her internet bill | |  | 0 | |
| - Cook |  | 1 | - Her rent | |  | 1 | |
| - Cashier |  | 0 | - Her bus pass | |  | 0 | |
| 5. Where did she work? |  |  | 12. How many days had it been since they had last eaten? | |  |  | |
| - In a daycare |  | 0 | - half a day | |  | 0 | |
| - In a canteen |  | 0 | - 2 days | |  | 1 | |
| - In a school |  | 1 | - 36 hours | |  | 0 | |
| 6. What happened to her? |  |  | 13. What did the police do? | |  |  | |
| - She was robbed |  | 1 | - Warn the media | |  | 0 | |
| - She broke her leg |  | 0 | - Took up a collection for her | |  | 1 | |
| - She got lost |  | 0 | - Investigated | |  | 0 | |
| 7. When was she robbed? |  |  |  | |  |  | |
| - The night before |  | 1 |  |  |  |  | |
| - Wednesday |  | 0 |  |  |  |  | |
| - This morning |  | 0 |  |  |  |  | |
|  |  |  | Total cued recall  = /13 | Total multiple choices = | | | /13 |

| # Question | Spont. | Choice | # Question | | Spont. | Choice | |
| --- | --- | --- | --- | --- | --- | --- | --- |
| 1. What was the man’s first name? |  |  | 8. When was it supposed to start? | |  |  | |
| - Joe |  | 1 | - Around 11 pm | |  | 0 | |
| - Jack |  | 0 | - The next day | |  | 0 | |
| - Jared |  | 0 | - In 2 to 3 hours | |  | 1 | |
| 2. What was the man’s last name? |  |  | 9. Until when was this special weather forecast? | |  |  | |
| - Scott |  | 0 | - Until the morning | |  | 1 | |
| - Smith |  | 1 | - Until Tuesday afternoon | |  | 0 | |
| - Stevens |  | 0 | - Until dinnertime | |  | 0 | |
| 3. Where did he live? |  |  | 10. How many inches of rain were expected? | |  |  | |
| - Sherbrooke |  | 1 | - 3 inches | |  | 0 | |
| - St-Lambert |  | 0 | - 5 inches | |  | 1 | |
| - Beloeil |  | 0 | - 6 inches | |  | 0 | |
| 4. What day of the week was it? |  |  | 11. By how many degrees was the temperature supposed to drop? | |  |  | |
| - Thursday |  | 0 | - 20 degrees | |  | 0 | |
| - Saturday |  | 0 | - 7 degrees | |  | 0 | |
| - Monday |  | 1 | - 15 degrees | |  | 1 | |
| 5. What time was it? |  |  | 12. Other than rain and a drop in temperature, what is the forecast? | |  |  | |
| - 6 pm |  | 1 | - Hail | |  | 1 | |
| - 7 pm |  | 0 | - Ice storms | |  | 0 | |
| - 9 pm |  | 0 | - A smog alert | |  | 0 | |
| 6. What did he do while watching television before the weather report? |  |  | 13. What did the man decide to do? | |  |  | |
| - Cleaning the house |  | 0 | - Close your windows | |  | 0 | |
| - Getting dressed |  | 1 | - Turn up the heat | |  | 0 | |
| - Looking out the window |  | 0 | - Staying at home | |  | 1 | |
| 7. What did the weather bulletin say? |  |  | 14. What did he watch on television? | |  |  | |
| - A thunderstorm |  | 0 | - A comedy | |  | 0 | |
| - A hurricane |  | 1 | - A documentary | |  | 0 | |
| - A tornado |  | 0 | - Old movies | |  | 1 | |
|  |  |  | Total cued recall  = /14 | Total multiple choices = | | | /14 |

*Note.* 1. The examiner must change an answer choice if the participant gives an incorrect response that is part of the answer choices.

**WMS-III-LM Recognition Procedure at Two Weeks – French Version**

***Story A and B^1^***

| # Question | Spont. | Choix | # Question | | Spont. | Choix |
| --- | --- | --- | --- | --- | --- | --- |
| 1. Quel était le prénom de la femme ? |  |  | 8. Combien d’argent lui a-t-on volé ? | |  |  |
| - Johanne |  | 0 | - 42 $ |  |  | 0 |
| - Jeanne |  | 1 | - 56 $ |  |  | 1 |
| - Julie |  | 0 | - 75 $ |  |  | 0 |
| 2. Quel était le nom de famille de la femme ? |  |  | 9. Sur quelle rue a eu lieu le vol ? | |  |  |
| - Dorval |  | 0 | - Sherbrooke |  |  | 0 |
| - Dupré |  | 0 | - Ste-Catherine |  |  | 1 |
| - Duval |  | 1 | - St-Joseph |  |  | 0 |
| 3. D’où venait-elle ? |  |  | 10. Combien avait-elle d’enfants ? | |  |  |
| - Montréal-Est |  | 1 | - 1 enfant |  |  | 0 |
| - Montréal-Nord |  | 0 | - 3 enfants |  |  | 0 |
| - Longueuil |  | 0 | - 4 enfants |  |  | 1 |
| 4. Que faisait-elle comme travail ? |  |  | 11. Qu’est-ce qui n’était pas payé ? | |  |  |
| - Caissière |  | 0 | - Sa passe d'autobus |  |  | 0 |
| - Cuisinière |  | 1 | - Son loyer |  |  | 1 |
| - Éducatrice |  | 0 | - Sa facture d'internet |  |  | 0 |
| 5. Où travaillait-elle ? |  |  | 12. Depuis combien de temps n’avaient-ils pas mangé ? |  |  |  |
| - Dans une cantine |  | 0 | - une demi-journée |  |  | 0 |
| - Dans une garderie |  | 0 | - 2 jours |  |  | 1 |
| - Dans une école |  | 1 | - 36 heures |  |  | 0 |
| 6. Que lui est-il arrivé ? |  |  | 13. Qu’on fait les policiers ? |  |  |  |
| - Elle a été volée |  | 1 | - Fait une enquête |  |  | 0 |
| - Elle s'est cassé une jambe |  | 0 | - Donné de l’argent à la femme | |  | 1 |
| - Elle s'est perdue |  | 0 | - Averti les médias |  |  | 0 |
| 7. Quand a-t-elle été volée ? |  |  |  | |  |  |
| - La veille |  | 1 |  |  |  |  |
| - Mercredi |  | 0 |  |  |  |  |
| - Ce matin |  | 0 |  |  |  |  |
|  | | | Total rappel indicé  = /13 | Total choix  multiples = | | /13 |

| # Question | Spont. | Choix | # Question | | Spont. | Choix |
| --- | --- | --- | --- | --- | --- | --- |
| 1. Quel était le prénom de l'homme ? |  |  | 8. Quand cela devait-il commencer ? | |  |  |
| - Pierre |  | 1 | - Vers 23h |  |  | 0 |
| - Philippe |  | 0 | - Le lendemain |  |  | 0 |
| - Pascal |  | 0 | - Dans 2 à 3 heures |  |  | 1 |
| 2. Quel était le nom de famille de l'homme? |  |  | 9. Jusqu'à quand cette météo spéciale était-elle prévue ? | |  |  |
| - Giguère |  | 0 | - Jusqu'au matin |  |  | 1 |
| - Gagnon |  | 1 | - Jusqu'à mardi après-midi |  |  | 0 |
| - Dufour |  | 0 | - Jusqu'à l'heure du dîner |  |  | 0 |
| 3. Où habitait-il ? |  |  | 10. Combien de cm de pluie étaient prévus ? | |  |  |
| - St-Hyacinthe |  | 1 | - 8 cm |  |  | 0 |
| - St-Lambert |  | 0 | - 10 cm |  |  | 1 |
| - Beloeil |  | 0 | - 12 cm |  |  | 0 |
| 4. Quel jour de la semaine se passe l'histoire ? |  |  | 11. Combien de degrés chuterait la température ? | |  |  |
| - Samedi |  | 0 | - 25 degrés |  |  | 0 |
| - Jeudi |  | 0 | - 20 degrés |  |  | 0 |
| - Lundi |  | 1 | - 15 degrés |  |  | 1 |
| 5. Quelle heure était-il ? |  |  | 12. Autre que la pluie et la baisse de température, qu'est-il annoncé  d'autre ? | |  |  |
| - 6h00 |  | 1 | - De la grêle |  |  | 1 |
| - 7h00 |  | 0 | - Du verglas |  |  | 0 |
| - 9h00 |  | 0 | - Une alerte de smog |  |  | 0 |
| 6. Que faisait-il en regardant la télévision avant le bulletin météo ? |  |  | 13. Que décida-t-il de faire ? |  |  |  |
| - Il faisait le ménage |  | 0 | - De fermer ses fenêtres |  |  | 0 |
| - Il s'habillait |  | 1 | - Monter le chauffage |  |  | 0 |
| - Il regardait par la fenêtre |  | 0 | - Rester à la maison |  |  | 1 |
| 7. Qu'annonçait le bulletin météo ? |  |  | 14. Qu'a-t-il regardé à la télévision ? | |  |  |
| - Un ouragan |  | 0 | - Un reportage |  |  | 0 |
| - Un orage |  | 1 | - Un téléroman |  |  | 0 |
| - Une tornade |  | 0 | - Un film |  |  | 1 |
|  | | | Total rappel indicé  = /14 | Total choix  multiples = | | /14 |

*Note.* 1. The examiner must change an answer choice if the participant gives an incorrect response that is part of the answer choices.

**RAVLT Recognition Procedure at 30 Minutes – English Version**

|  | **Y** | **N** |  | **Y** | **N** |  | **Y** | **N** |
| --- | --- | --- | --- | --- | --- | --- | --- | --- |
| Rock (PB) |  |  | Hospital (SB) |  |  | Bucket (A) |  |  |
| Corn (PB) |  |  | Grass (SA) |  |  | Soldier (B) |  |  |
| Pear (SA) |  |  | Lunchbox (SA) |  |  | Scarf (A) |  |  |
| Tree (A) |  |  | Soap (B) |  |  | Wife (SA) |  |  |
| Cork (B) |  |  | Frog (SB) |  |  | Train (SB) |  |  |
| Bread |  |  | Dog (A) |  |  | Suitcase (A) |  |  |
| Sofa (SB) |  |  | Orange (B) |  |  | Violin (A) |  |  |
| Stair (A) |  |  | Cousin (A) |  |  | Beach (B) |  |  |
| Ham (A) |  |  | Bathroom (B) |  |  | Radio (A) |  |  |
| Casserole (B) |  |  | Gloves (SA) |  |  | Bus (B) |  |  |
| Star (SA) |  |  | Field (A) |  |  | Doctor |  |  |
| Peel (SA) |  |  | Armchair (B) |  |  | Chest |  |  |
| Lock (B) |  |  | Blanket (PA) |  |  | Knife (A) |  |  |
| Banana (A) |  |  | Television (SA) |  |  | Donkey (B) |  |  |
| Toad (B) |  |  | Hotel (B) |  |  | Hunter (A) |  |  |
| Uncle (SA) |  |  | Piano (SA) |  |  | Chin (B) |  |  |
| Earth (A) |  |  | Spider (B) |  |  |  |  |  |
|  |  |  |  |  |  |  |  |  |
| *Number of words from List A recognized* | | | | | | */ 15* | | |
| *Number of words from List A forgotten* | | | | | |  | | |
| *Number of false positives* | | | | | | */ 35* | | |

**RAVLT Recognition Procedure at Two Weeks – English Version**

|  | **Y** | **N** |  | **Y** | **N** |  | **Y** | **N** |
| --- | --- | --- | --- | --- | --- | --- | --- | --- |
| Garden (SA) |  |  | Trunk (SA) |  |  | Stair (A) |  |  |
| Duck (PB) |  |  | Banana (A) |  |  | Orange (B) |  |  |
| Violin (A) |  |  | Hotel (B) |  |  | Toad (B) |  |  |
| Knob (SB) |  |  | Radio (A) |  |  | Plane (SB) |  |  |
| Scarf (A) |  |  | Elevator (SA) |  |  | Chin (B) |  |  |
| Soldier (B) |  |  | Bucket (A) |  |  | Hunter (A) |  |  |
| Ham (A) |  |  | Field (A) |  |  | Soap (B) |  |  |
| Apple (SA) |  |  | Beach (B) |  |  | Insect (SB) |  |  |
| Suitcase (A) |  |  | Chicken (SA) |  |  | Tree (A) |  |  |
| Stereo (SA) |  |  | Classroom (PB) |  |  | Bus (B) |  |  |
| Armchair (B) |  |  | Rabbit (SA) |  |  | Bathroom (B) |  |  |
| Cousin (A) |  |  | Agenda |  |  | Dog (A) |  |  |
| Chair (SB) |  |  | Donkey (B) |  |  | Door |  |  |
| Spoon (SA) |  |  | Knife (A) |  |  | Casserole (B) |  |  |
| Shield (PA) |  |  | Spider (B) |  |  | Lock (B) |  |  |
| Earth (A) |  |  | Guitar (SA) |  |  | Pharmacy |  |  |
| Cork (B) |  |  | Son (SB) |  |  |  |  |  |
|  |  |  |  |  |  |  |  |  |
| *Number of words from List A recognized* | | | | | | */ 15* | | |
| *Number of words from List A forgotten* | | | | | |  | | |
| *Number of false positives* | | | | | | */ 35* | | |

**RAVLT Recognition Procedure at 30 Minutes – French Version**

|  | **Y** | **N** |  | **Y** | **N** |  | **Y** | **N** |
| --- | --- | --- | --- | --- | --- | --- | --- | --- |
| Fourrure (PB) |  |  | Hôpital (SB) |  |  | Seau (A) |  |  |
| Bouillon (PB) |  |  | Herbe (SA) |  |  | Soldat (B) |  |  |
| Poire (SA) |  |  | Boîte (SA) |  |  | Cravate (A) |  |  |
| Arbre (A) |  |  | Savon (B) |  |  | Femme (SA) |  |  |
| Bouchon (B) |  |  | Grenouille (SB) |  |  | Autobus (SB) |  |  |
| Pain |  |  | Chien (A) |  |  | Valise (A) |  |  |
| Sofa (SB) |  |  | Orange (B) |  |  | Violon (A) |  |  |
| Escalier (A) |  |  | Cousin (A) |  |  | Rivage (B) |  |  |
| Jambon (A) |  |  | Toilette (B) |  |  | Outil (A) |  |  |
| Marmite (B) |  |  | Habit (SA) |  |  | Train (B) |  |  |
| Bouche (SA) |  |  | Campagne (A) |  |  | Docteur |  |  |
| Écorce (SA) |  |  | Fauteuil (B) |  |  | Poitrine |  |  |
| Serrure (B) |  |  | Peau (PA) |  |  | Couteau (A) |  |  |
| Banane (A) |  |  | Fourchette (SA) |  |  | Cheval (B) |  |  |
| Crapaud (B) |  |  | Hôtel (B) |  |  | Chasseur (A) |  |  |
| Oncle (SA) |  |  | Piano (SA) |  |  | Menton (B) |  |  |
| Oreille (A) |  |  | Insecte (B) |  |  |  |  |  |
|  |  |  |  |  |  |  |  |  |
| *Nombre de mots de la liste A reconnus* | | | | | | */ 15* | | |
| *Nombre de fausses reconnaissances* | | | | | | */ 35* | | |
| *Fausses reconnaissances – Liste B* | | | | | | */ 15* | | |

**RAVLT Recognition Procedure at Two Weeks – French Version**

|  | **Y** | **N** |  | **Y** | **N** |  | **Y** | **N** |
| --- | --- | --- | --- | --- | --- | --- | --- | --- |
| Champ (SA) |  |  | Tronc (SA) |  |  | Escalier (A) |  |  |
| Rayure (PB) |  |  | Banane (A) |  |  | Orange (B) |  |  |
| Violon (A) |  |  | Hôtel (B) |  |  | Crapaud (B) |  |  |
| Poignée (SB) |  |  | Outil (A) |  |  | Avion (SB) |  |  |
| Cravate (A) |  |  | Ascenseur (SA) |  |  | Menton (B) |  |  |
| Soldat (B) |  |  | Seau (A) |  |  | Chasseur (A) |  |  |
| Jambon (A) |  |  | Campagne (A) |  |  | Savon (B) |  |  |
| Pomme (SA) |  |  | Rivage (B) |  |  | Araignée (SB) |  |  |
| Valise (A) |  |  | Poulet (SA) |  |  | Arbre (A) |  |  |
| Scie (SA) |  |  | Tablette (PB) |  |  | Train (B) |  |  |
| Fauteuil (B) |  |  | Lapin (SA) |  |  | Toilette (B) |  |  |
| Cousin (A) |  |  | Auberge |  |  | Chien (A) |  |  |
| Chaise (SB) |  |  | Cheval (B) |  |  | Porte |  |  |
| Cuillère (SA) |  |  | Couteau (A) |  |  | Marmite (B) |  |  |
| Champagne (PA) |  |  | Insecte (B) |  |  | Serrure (B) |  |  |
| Oreille (A) |  |  | Guitare (SA) |  |  | Pharmacie |  |  |
| Bouchon (B) |  |  | Fils (SA) |  |  |  |  |  |
|  |  |  |  |  |  |  |  |  |
| *Nombre de mots de la liste A reconnus* | | | | | | */ 15* | | |
| *% Rétention* | | | | | |  | | |
| *Nombre de fausses reconnaissances* | | | | | | */ 35* | | |
| *Fausses reconnaissances – Liste B* | | | | | | */ 15* | | |

**Figure S1**

*Distribution of RAVLT (Fig. S1A) and WMS-III-LM scores (Fig. S1B) among health participants (n = 124).* *White dot represents the median, and black box encompasses the data between the 25th and 75th percentiles*. *The x-axis represents the density of the data distribution.*


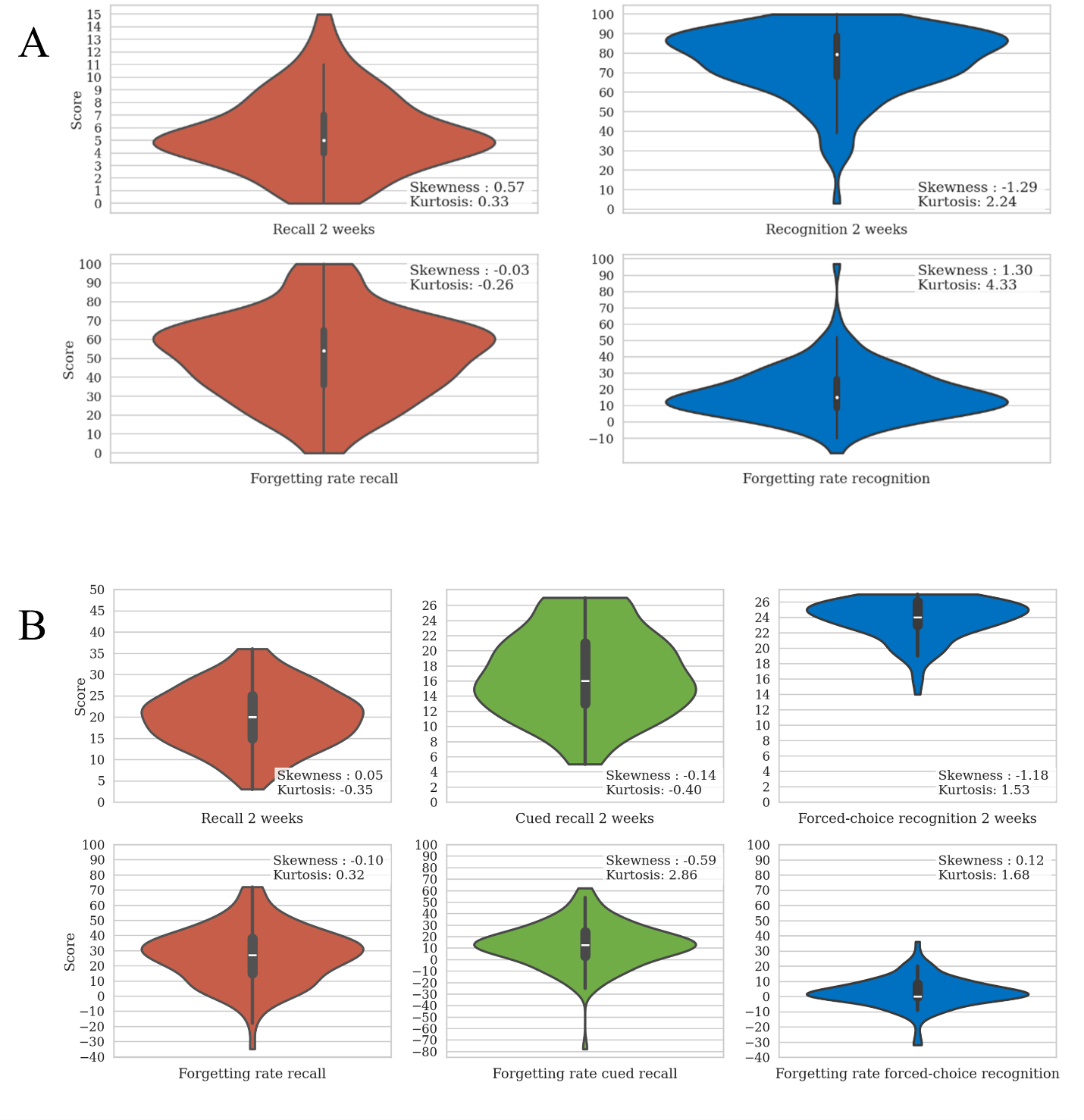


**Table S1**

| Memory scores | Age | Sex | Education | Ethnicity | BNT | WAIS-IV-MR | Mode of testing |
| --- | --- | --- | --- | --- | --- | --- | --- |
| RAVLT score |  |  |  |  |  |  |  |
| Ʃ Trials 1-5 | -0.28** | 0.24** | 0.16† | -0.06 | 0.085 | 0.157 | 1.087 |
| Immediate recall | -0.31** | 0.22* | 0.04 | -0.03 | 0.017 | 0.138 | 0.139 |
| 30-min. delayed recall | -0.38** | 0.29** | 0.05 | -0.03 | 0.037 | 0.139 | 0.004 |
| 30-min. delayed recognition | -0.36** | 0.14 | 0.11 | 0.05 | -0.065 | 0.120 | 1.014 |
| 2-week delayed recall | -0.26** | 0.14 | 0.01 | -0.10 | 0.049 | 0.090 | 0.549 |
| 2-week delayed recognition | -0.31** | 0.20* | 0.05 | -0.09 | -0.036 | 0.158 | 0.335 |
| Forgetting rate recall | 0.11 | -0.01 | 0.03 | 0.10 | -0.066 | -0.029 | 1.066 |
| Forgetting rate recognition | 0.16† | -0.17† | -0.02 | 0.15 | -0.055 | -0.189* | 0.41 |
| WMS-III-LM score |  |  |  |  |  |  |  |
| Immediate recall | -0.27** | 0.23* | 0.20* | 0.02 | 0.033 | 0.198* | 0.107 |
| 30-min. delayed recall | -0.24** | 0.26** | 0.20* | 0.04 | 0.088 | 0.169 | 0.371 |
| 30-min. delayed cued recall | -0.14 | 0.27** | 0.20* | -0.01 | 0.043 | 0.177 | 0.296 |
| 30-min. delayed forced-choice recognition | -0.07 | 0.19* | 0.19* | -0.06 | 0.013 | 0.183* | 1.164 |
| 2-week delayed recall | -0.15† | 0.21* | 0.21* | -0.06 | 0.096 | 0.176 | 0.162 |
| 2-week delayed cued recall | -0.08 | 0.27** | 0.13 | -0.07 | 0.124 | 0.142 | 0.165 |
| 2-week delayed forced-choice recognition | -0.03 | 0.29** | 0.07 | -0.17† | 0.141 | 0.134 | 1.499 |
| Forgetting rate recall | -0.05 | -0.02 | -0.07 | 0.16† | -0.038 | -0.159 | 0.121 |
| Forgetting rate cued recall | -0.02 | -0.04 | 0.07 | 0.14 | -0.118 | -0.086 | 0.280 |
| Forgetting rate forced-choice recognition | -0.02 | -0.13 | 0.08 | 0.08 | -0.132 | -0.018 | 1.714 |

*Associations between sociodemographic, complementary neuropsychological scores, self-administered questionnaires, and mode of testing, and memory scores among health participants (n = 124)*

*Note.* Values are Pearson correlation coefficients, except for correlations involving education, BNT, WAIS-IV-MR, RAVLT recognition scores, 2-week delayed forced-choice recognition, and forgetting rate for cued recall and forced-choice recognition in the WMS-III-LM (Spearman correlations). The correlations were completed with raw scores except for the WAIS-IV-MR, where scaled scores were taken. The mode of testing displays F values with 2 degrees of freedom between groups and 121 within groups; * *p* < 0.05; ** *p* < 0.01; † p < 0.10.

**Table S2**

*Mean (SD) performance for additional WMS-III-LM variables among healthy participants*

| WMS-III-LM score | | | Male (n = 61) | | |  | Female (n = 63) | | |
| --- | --- | --- | --- | --- | --- | --- | --- | --- | --- |
|  |  |  | 18-29 (n=20) | 30-41 (n=19) | 42-55 (n=21) |  | 18-29 (n=23) | 30-41 (n=18) | 42-55 (n=22) |
| Story A (/25) | | | 15.60 (3.63) | 15.95 (3.97) | 13.95 (2.94) |  | 16.24 (3.57) | 16.56 (3.96) | 15.10 (4.45) |
| Story B1 (/25) | | | 10.85 (3.66) | 11.53 (4.38) | 10.10 (2.51) |  | 13.52 (3.72) | 13.17 (3.29) | 11.24 (3.59) |
| Story B2 (/25) | | | 16.10 (2.94) | 16.58 (4.25) | 14.24 (3.49) |  | 18.36 (3.00) | 17.83 (3.38) | 15.90 (4.55) |
|  | | |  |  |  |  |  |  |  |
| 30-min. recall story A (/25) | | | 13.10 (3.68) | 12.53 (5.41) | 11.33 (3.48) |  | 14.00 (4.72) | 14.50 (3.22) | 13.24 (4.07) |
| 30-min. recall story B (/25) | | | 14.10 (3.96) | 14.32 (3.79) | 10.86 (4.11) |  | 16.04 (3.96) | 15.72 (3.98) | 14.10 (4.55) |
| 30-min. recognition | | |  |  |  |  |  |  |  |
|  | Cued recall story A (/13) | | 7.45 (2.63) | 7.84 (2.43) | 7.05 (1.99) |  | 8.06 (2.65) | 8.83 (1.51) | 8.33 (2.74) |
|  | Cued recall story B (/14) | | 10.20 (2.22) | 10.84 (2.67) | 9.43 (2.70) |  | 11.60 (1.92) | 11.61 (1.75) | 10.52 (2.68) |
|  | Total story A (/13) † | | 11.35 (1.50) | 11.32 (1.11) | 11.33 (1.59) |  | 11.76 (1.27) | 12.00 (1.03) | 11.76 (1.18) |
|  | Total story B (/14) | | 12.85 (1.27) | 13.05 (1.31) | 12.52 (1.37) |  | 13.00 (1.19) | 13.22 (0.94) | 13.05 (0.97) |
| 2-week delayed recall story A (/25) | | | 9.55 (3.82) | 9.11 (4.88) †† | 8.71 (3.52) |  | 11.32 (3.98) | 10.56 (3.22) | 11.05 (3.76) |
| 2-week delayed recall story B (/25) | | | 9.95 (3.35) | 9.95 (4.98) †† | 8.43 (4.12) |  | 11.32 (4.31) | 10.11 (4.00) | 9.90 (4.06) |
| 2-week recognition | | |  |  |  |  |  |  |  |
|  | | Cued recall story A (/13) | 6.75 (2.57) | 6.79 (2.76) | 6.52 (2.32) |  | 7.80 (2.36) | 7.44 (1.46) | 7.24 (2.79) |
|  | | Cued recall story B (/14) | 8.35 (2.58) | 8.63 (3.10) | 7.29 (2.47) |  | 9.52 (2.38) | 9.44 (2.53) | 9.14 (2.99) |
|  | | Total story A (/13) † | 10.60 (1.60) | 10.63 (2.36) | 10.33 (1.68) |  | 11.48 (1.36) | 11.00 (1.46) | 11.24 (1.76) |
|  | | Total story B (/14) † | 12.55 (1.43) | 12.58 (1.39) | 12.48 (1.40) |  | 13.24 (0.88) | 12.89 (1.02) | 13.24 (1.90) |

*Note.* † Variables non-normally distributed; ††Variables that cannot get at least 2 SD below the average.

**Table S3**

| RAVLT score | Coefficients: *β*, *p* | F, *p* | R^2^ | Adj. R^2^ | Equation |
| --- | --- | --- | --- | --- | --- |
| Trial 1 | Sex: 0.699, *p* = 0.038  Age: -0.039, *p* = 0.010 | 5.873  *p =* 0.004 | 0.088 | 0.073 | Z = (Actual score – (6.506 + 0.699 S – 0.039 A)) / 1.848 |
| Trial 2 | Sex: 0.667, *p* = 0.127  Age: -0.041, *p* = 0.038 | 3.492  *p =* 0.034 | 0.055 | 0.039 | Z = (Actual score – (9.829 + 0.667 S – 0.041 A)) / 2.418 |
| Trial 3 | Sex: 0.874, *p* = 0.020  Age: -0.042, *p* = 0.013 | 6.222  *p* = 0.003 | 0.093 | 0.078 | Z = (Actual score – (11.584 + 0.874 S – 0.042 A)) / 2.060 |
| Trial 4 | Sex: 1.152, *p* = 0.001  Age: -0.044, *p* = 0.005 | 9.976  *p <* 0.001 | 0.142 | 0.127 | Z = (Actual score – (12.148 + 1.152 S – 0.044 A)) / 1.915 |
| Trial 5 | Sex: 0.560, *p* = 0.071  Age: -0.042, *p* = 0.003 | 6.617  *p* = 0.002 | 0.099 | 0.084 | Z = (Actual score – (13.443 + 0.560 S – 0.042 A)) / 1.707 |
| Ʃ Trials 1-5 | Sex: 3.952, *p* = 0.008  Age: -0.209, *p* = 0.002 | 8.876  *p <* 0.001 | 0.128 | 0.114 | Z = (Actual score – (53.509 + 3.952 S – 0.209 A)) / 8.198 |
| List B | Sex: 0.876, *p* = 0.027  Age: -0.077, *p* < 0.001 | 12.448  *p* < 0.001 | 0.171 | 0.157 | Z = (Actual score – (7.087 + 0.876 S – 0.077 A)) / 2.172 |
| Immediate recall | Sex: 1.115, *p* = 0.013  Age: -0.073, *p* < 0.001 | 10.133  *p <*0.001 | 0.143 | 0.126 | Z = (Actual score – (12.185 + 1.115 S – 0.073 A)) / 2.467 |
| 30-min. delayed recall | Sex: 1.519, *p* = 0.001  Age: -0.079, *p <* 0.001 | 13.475  *p <* 0.001 | 0.182 | 0.169 | Z = (Actual score – (11.698 + 1.519 S – 0.079 A)) / 2.530 |
| 2-week delayed recall | Sex: 0.811, *p* = 0.130  Age: -0.069, *p* = 0.005 | 5.485  *p* = 0.005 | 0.083 | 0.068 | Z = (Actual score – (6.760 + 0.811 S – 0.069 A)) / 2.960 |
| Forgetting rate recall | Delay: 1.734, *p* = 0.014 | 6.168  *p =* 0.014 | 0.048 | 0.040 | Z = (Actual score – (27.747 + 1.734 D)) / 22.091 |

| WMS-III-LM score | | Coefficients: *β*, *p* | F, *p* | R^2^ | | Adj. R^2^ | Equation |
| --- | --- | --- | --- | --- | --- | --- | --- |
| Total recall (A+B1+B2) | | Sex: 4.384, *p* = 0.009  Age: 1.000, *p* = 0.066  Age^2^: -0.017, *p* = 0.023 | 7.531  *p* < 0.001 | 0.158 | | 0.137 | Z = (Actual score – (25.583 + 4.384 S + 1.000 A – 0.017 A^2^)) / 9.124 |
| Recall first trial (A+B1) | | Sex: 2.626, *p* = 0.015  Age: 0.635, *p* = 0.088  Age^2^: -0.011, *p* = 0.037 | 5.849  *p* < 0.001 | 0.128 | | 0.106 | Z = (Actual score – (15.793 + 2.626 S + 0.635 A – 0.011 A^2^)) / 6.264 |
| 30-min. delayed recall (A+B) | | Sex: 3.742, *p* = 0.004  Age: -0.158, *p* = 0.007 | 8.351  *p* < 0.001 | 0.121 | | 0.107 | Z = (Actual score – (27.196 + 3.742 S – 0.158 A)) / 7.130 |
| 30-min. recognition | | |  |  |  | |  |
|  | Cued recall (A + B) | Sex: 1.086, *p* = 0.011  Age: -0.038, *p* = 0.049 | 5.475  *p* = 0.005 | 0.083 | | 0.068 | Z = (Actual score – (15.305 + 2.261 S)) / 4.087 |
|  | Forced-choice (A + B) | Sex: 0.763, *p* = 0.043  Age: -0.013, *p* = 0.449 | 2.434  *p* = 0.092 | 0.039 | | 0.023 | Z = (Actual score – (23.360 + 0.773 S)) / 2.073 |
| 2-week delayed recall (A + B) | | Sex: 2.921, *p* = 0.024  Age: -0.096, *p* = 0.098 | 4.141  *p* = 0.018 | 0.064 | | 0.049 | Z = (Actual score – (15.535 + 2.998 S)) / 7.169 |
| 2-week recognition | | |  |  | |  |  |
|  | Cued recall (A + B) | Sex: 2.921, *p* = 0.024  Age: -0.096, *p* = 0.098 | 4.141  *p* = 0.018 | 0.064 | | 0.049 | Z = (Actual score – (12.266 + 2.484 S)) / 4.520 |
| Forgetting rate recall | | Delay: 1.835, *p* = 0.004 | 8.637  *p =* 0.004 | 0.066 | | 0.058 | Z = (Actual score – (1.393 + 1.835 D)) / 19.760 |

*Summary of the linear regression for each test variable (from health participants (n = 124))*

*Note.* A = age in years; A^2^ = age in years squared; D = delay in days; S = sex (1 = men, 2 = women).
