# Supplementary material for: Adaptation of the Rey Auditory Verbal Learning Test and Logical Memory Subtest from the Wechsler Memory Scales – 3rd Edition to assess accelerated long‐term forgetting in adults with epilepsy
Source: Epileptic Disord. 2025 Aug 14;27(6):1187–200. doi: 10.1002/epd2.70084 (PMC12747703; doi:10.1002/epd2.70084)
Supplement: Supplementary file 2 — Data S2. [file EPD2-27-1187-s001.docx]

Answers

1. B

2. D

3. A
